# Shoenfeld syndrome or Autoimmune/Inflammatory Syndrome Induced by Adjuvants syndrome: a case of inguinal hernia mesh rejection after 9 years

**DOI:** 10.1093/jscr/rjag120

**Published:** 2026-04-30

**Authors:** Sarah Benammi, Imane Joutei Boutaleb, Bensaad Ahmed, Nouri Abdellah, Ghaddou Youssef, Mohamed Bouziane, Sair Khalid, Fadil Abdelaziz

**Affiliations:** Mohammed VI University of Sciences and Health, Ave Mohamed Taieb Naciri, Casablanca, Morocco; International University Hospital Mohammed VI, Ave Mohamed Taieb Naciri, Casablanca 82403, Morocco; Mohammed VI University of Sciences and Health, Ave Mohamed Taieb Naciri, Casablanca, Morocco; International University Hospital Cheikh Khalifa, P3011, Bouskoura 27182, Casablanca, Morocco; Mohammed VI University of Sciences and Health, Ave Mohamed Taieb Naciri, Casablanca, Morocco; International University Hospital Mohammed VI, Ave Mohamed Taieb Naciri, Casablanca 82403, Morocco; Mohammed VI University of Sciences and Health, Ave Mohamed Taieb Naciri, Casablanca, Morocco; International University Hospital Cheikh Khalifa, P3011, Bouskoura 27182, Casablanca, Morocco; Mohammed VI University of Sciences and Health, Ave Mohamed Taieb Naciri, Casablanca, Morocco; International University Hospital Mohammed VI, Ave Mohamed Taieb Naciri, Casablanca 82403, Morocco; Mohammed VI University of Sciences and Health, Ave Mohamed Taieb Naciri, Casablanca, Morocco; International University Hospital Cheikh Khalifa, P3011, Bouskoura 27182, Casablanca, Morocco; Mohammed VI University of Sciences and Health, Ave Mohamed Taieb Naciri, Casablanca, Morocco; International University Hospital Cheikh Khalifa, P3011, Bouskoura 27182, Casablanca, Morocco; Mohammed VI University of Sciences and Health, Ave Mohamed Taieb Naciri, Casablanca, Morocco; International University Hospital Cheikh Khalifa, P3011, Bouskoura 27182, Casablanca, Morocco

**Keywords:** Shoenfeld syndrome, ASIA syndrome, inguinal hernia, foreign body rejection, mesh rejection

## Abstract

Autoimmune/Inflammatory Syndrome Induced by Adjuvants (ASIA Syndrome), also called Schoenfeld syndrome, is a rare inflammatory reaction to implant materials. We report a case of mesh rejection nine years after left inguinal hernia repair in a 62-year-old man presenting with intense localized pain and inflammatory signs, including psoasitis. Imaging revealed sepsis with an inguinal collection extending to the ipsilateral iliopsoas and sartorius muscles. The patient underwent surgical mesh removal and drainage; tissue samples showed aseptic fluid collections without pathogens. Postoperatively, the patient received four sessions of hyperbaric oxygen therapy alongside radiological drainage of a residual collection in the lower limb. Literature on inflammatory responses to mesh rejection remains scarce, with ASIA Syndrome potentially triggered by any implant and with uncertain prognosis. Delayed mesh rejection is a notable complication of hernia repair, warranting patient awareness. Treatment includes mesh extraction, inflammation control, drainage, and hyperbaric therapy, though management guidelines remain undefined.

## Introduction

In recent years, the use of mesh has become a standard procedure in hernia repair surgery worldwide, with a notable reduction in hernia recurrence rates [1]. However, mesh-related complications have become increasingly significant and may develop years after implantation [1, 2]. Advances in technology have produced relatively inert and biocompatible meshes, but these implants can still trigger adverse reactions, including inflammation [foreign body reaction (FBR)], fibrosis, calcification, thrombosis, and infection [1]. This inflammatory response to implant materials is known as ‘Autoimmune/Inflammatory Syndrome Induced by Adjuvants’ (ASIA Syndrome) or Shoenfeld syndrome [3]. There is scarce data on Shoenfeld syndrome linked to hernia mesh, and its management remains poorly defined.

We report a case of aseptic polypropylene mesh rejection nine years after Lichtenstein hernia repair and discuss our management approach in a university referral setting.

## Case report

**Figure 1:**
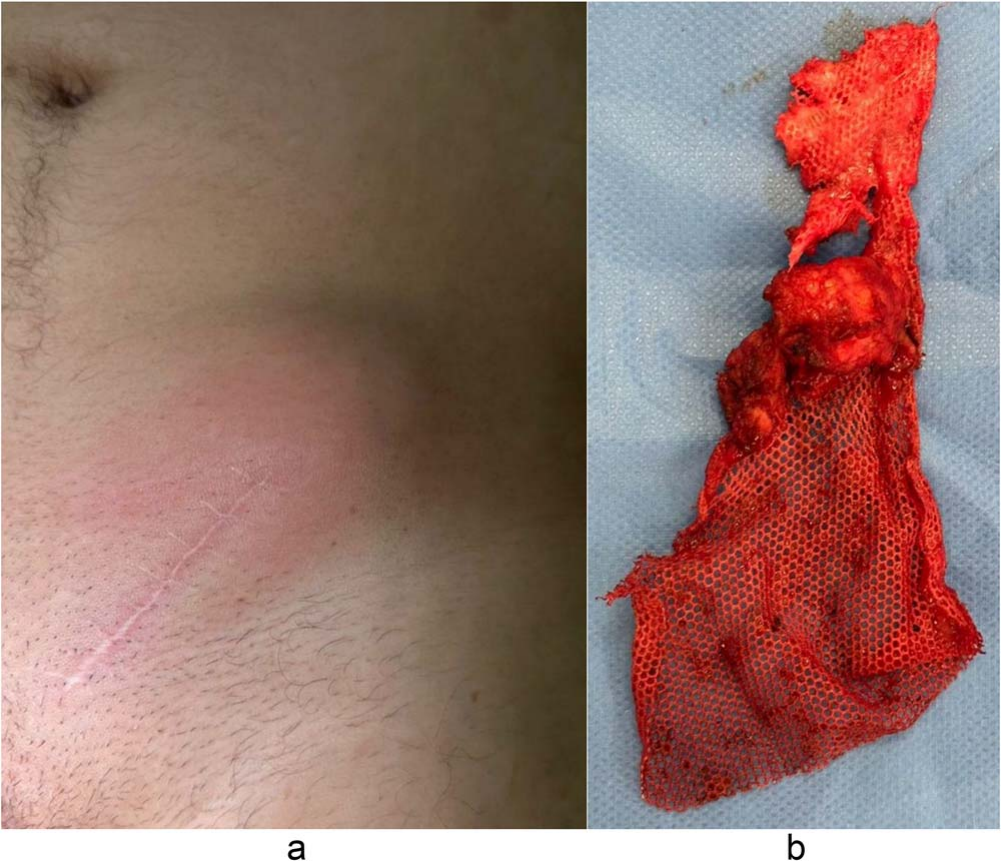
(a) Clinical symptoms of inflammation regarding inguinal hernia scar. (b) Removed hernia mesh after surgical intervention.

**Figure 2:**
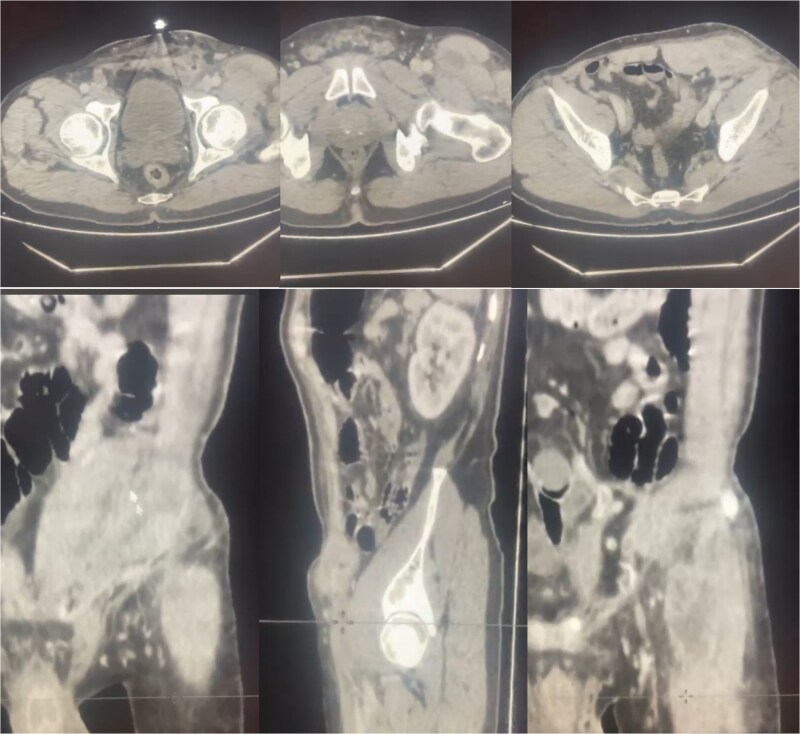
Abdominal pelvic CT-scan showing inflammation around the left inguinal mesh with infiltration and thickening around the adjacent parietal soft tissues and multiple collections surrounding the mesh and along the ipsilateral iliopsoas and sartorius muscles.

A 62-year-old North African Caucasian male with a BMI of 24 kg/m^2^ had a left inguinal hernia repaired in 2015 via Lichtenstein technique using polypropylene mesh. He had no autoimmune diseases or allergies and a history of left knee ligament tear. Four days before admission, he developed intense intermittent left inguinal pain. Physical exam showed the surgical scar, redness, swelling, tenderness, psoitis, and functional impairment of the left lower limb (Fig. 1a). The patient was febrile (39°C). Lab tests indicated sepsis with elevated C-reactive protein (114.9 mg/L) and leukocytosis (16 000 WBC/μL). Computed tomography (CT) scan revealed inflammation and multiple collections around the mesh extending into ipsilateral muscles (Fig. 2).

Medical treatment was initiated; antibiotics were withheld until sampling. Surgery included open removal of the mesh (Fig. 1b). The mesh was partially adherent to tissues; the other part was free in the pus. Complete mesh removal was performed. Reconstruction without mesh was done; the wound was partially closed for delayed dressing.

Collections in the upper left thigh were drained radiologically, with samples taken. The postoperative course was uncomplicated. The patient received four hyperbaric oxygen therapy sessions starting postoperative day one, alongside daily local wound care using alginate dressings inserted through a small wound opening. He was discharged on day five with home nursing until full wound healing by week three. Follow-up imaging showed no fluid collection or symptom recurrence. Microbiology and histology confirmed aseptic fluid and FBR without pathogens.

At two months, the patient developed right lower limb deep vein thrombosis, managed with anticoagulants. At six months, no left-side recurrence or inflammation was noted. A small right inguinal hernia was repaired laparoscopically using polypropylene mesh after informed consent considering rejection risk. Recovery was smooth with no inflammatory symptoms at two-month follow-up.

The report complies with SCARE criteria [4] and had institutional exemption from ethical approval. Written informed consent for publication was obtained. No funding or conflicts of interest were declared.

## Discussion

Hernia mesh complications, including infection and inflammatory reactions, cause significant morbidity, readmissions, healthcare costs, hernia recurrence, and lower quality of life [5]. FBR involves macrophage activation releasing inflammatory cytokines, potentially years after implantation [1]. Our patient’s clinical and biological findings aligned with foreign body inflammatory reaction, supporting an autoimmune inflammatory syndrome diagnosis. Such reactions manifest as seroma, rejection, mesh migration, adhesions, and pain. Few studies have explored inflammatory mesh rejection [6, 7].

Schachtrupp *et al*. [8] analyzed local inflammation induced by hernia mesh, especially cytokine release and monocyte roles, showing patient variability likely linked to individual susceptibility. Larger studies are necessary. This susceptibility to exaggerated immune responses corresponds to ASIA syndrome, first described in 2011 [8–10]. Diagnostic criteria for ASIA remain uncertain due to limited evidence. The diagnosis of ASIA requires two major criteria, or one major and two minor criteria: major criteria (exposure to external element (infection, vaccine, foreign body; presence of typical clinical manifestations; myalgia, myositis, or muscle weakness; arthralgia and/or arthritis; chronic fatigue, sleep disturbances; neurological manifestations; cognitive impairment, memory loss; pyrexia, dry mouth; improvement after removal of causing agent; typical biopsy of involved organs) and minor criteria (Autoantibodies or antibodies directed to the suspected adjuvant, Other clinical manifestations (i.e. irritable bowel syndrome), Specific HLA (i.e. HLA DRB1, HLA DQB1); Development of an autoimmune disease) [10, 11]. Regarding our case, we reported 3 major criterias. Polypropylene mesh inflammation should be considered in patients with febrile illness or abdominal wall inflammation after hernia repair [1]. Mesh infection rates range from 1%–8%, affected by comorbidities, mesh type, surgical technique, and infection prevention [1].

The 2023 review by Cohen Tervaert *et al*. [10] expanded ASIA syndrome definition to include all implanted medical devices, describing it as a heterogeneous disease triggered by allogenic substances in susceptible hosts. Various risk factors have been proposed. Individuals with a history of allergies face higher chances of ASIA following implant placement. Likewise, those with pre-existing autoimmune conditions or genetic family history of autoimmunity may develop symptoms post-silicone breast implantation. Notably, immunogenetic elements (e.g. HLA profiles) interact with lifestyle factors like tobacco use and excess weight to trigger device-related ASIA [10]. Furthermore, implanted biomaterials elicit a FBR, causing granulomatous inflammation. Protein adsorption rapidly recruits pro-inflammatory M1 macrophages, aided by mast cells and histamine, which sensitize TRPV1 nociceptors to provoke severe implant-site pain. Biofilms on devices and ‘danger signals’ from biomaterials further sustain chronic inflammation [10]. Explantation leading to symptom improvement is a defining criterion. Surgical removal of the causative material is central to management, though complete removal is sometimes impossible, as reported by Puerta Sarmiento *et al*. [12]. Immunomodulatory treatments are rarely documented [12]. We combined surgical explantation, radiological drainage, and hyperbaric oxygen therapy, leading to favorable outcomes supporting ASIA diagnosis.

Cohen Tervaert *et al*. [10] identified factors influencing explantation outcomes—smoking, BMI, implant material, autoimmune diseases, exposure duration, and reconstruction strategy. These are well-defined for breast implant ASIA but need study in hernia mesh context.

## Conclusion

Delayed hernia mesh rejection, part of ASIA syndrome, should be explained to patients preoperatively. Due to occurrence in a small patient subset, clinical data are limited. More studies are needed to guide diagnosis and management. Current approaches include implant removal, inflammation management, collection drainage, and hyperbaric therapy, as demonstrated here.

Our case highlights the complexity of mesh-related inflammatory syndrome management and the value of a multidisciplinary approach involving surgery, radiology, infectious disease, and hyperbaric medicine. This approach facilitated uneventful recovery and symptom resolution. However, prognosis remains uncertain and further research is essential to develop standardized diagnosis and treatment protocols for ASIA syndrome in hernia mesh patients.

## References

[1] Falagas ME, Kasiakou SK. Mesh-related infections after hernia repair surgery. Clin Microbiol Infect 2005;11:3–8. 10.1111/j.1469-0691.2004.01014.x 15649297

[2] Kallinowski F, Fortelny RH, Köckerling F et al. Editorial: mesh complications in hernia surgery. Front Surg 2022;9:841672. 10.3389/fsurg.2022.841672 35372469 PMC8974239

[3] Negin F, Huynh D, Khanmohammed Z et al. Patients with systemic reaction to their hernia mesh: an introduction to mesh implant. J Abdom Wall Surg 2023;2:10983. 10.3389/jaws.2023.10983 38312397 PMC10831643

[4] Sohrabi C, Mathew G, Maria N et al. The SCARE 2023 guideline: updating consensus Surgical CAse REport (SCARE) guidelines. Int J Surg Lond Engl 2023;109:1136. 10.1097/JS9.0000000000000373 PMC10389401 37013953

[5] Wilson RB, Farooque Y. Risks and prevention of surgical site infection after hernia mesh repair and the predictive utility of ACS-NSQIP. J Gastrointest Surg 2022;26:950–64. 10.1007/s11605-022-05248-6 35064459 PMC9021144

[6] Beets GL, van Mameren H, Go PMNYH. Long-term foreign-body reaction to preperitoneal polypropylene mesh in the pig. Hernia 1998;2:153–5. 10.1007/bf01569134

[7] Klinge U, Klosterhalfen B, Muller M et al. Foreign body reaction to meshes used for the repair of abdominal wall hernias. Eur J Surg 1999;165:665–73. 10.1080/11024159950189726 10452261

[8] Schachtrupp A, Klinge U, Junge K et al. Individual inflammatory response of human blood monocytes to mesh biomaterials. Br J Surg 2003;90:114–20. 10.1002/bjs.4023 12520586

[9] Watad A, Qaresma M, Bragazzi NL et al. The autoimmune/inflammatory syndrome induced by adjuvants (ASIA)/Shoenfeld’s syndrome: descriptive analysis of 300 patients from the international ASIA syndrome registry. Clin Rheumatol 2018;37:483–93. 10.1007/s10067-017-3748-9 28741088

[10] Tervaert JWC, Martinez-Lavin M, Jara LJ et al. Autoimmune/inflammatory syndrome induced by adjuvants (ASIA) in 2023. Autoimmun Rev 2023;22:103287. 10.1016/j.autrev.2023.103287 36738954

[11] Caldarelli M, Rio P, Giambra V et al. ASIA syndrome: state-of-the-art and future perspectives. Vaccines (Basel) 2024;12:1183. 10.3390/vaccines12101183 39460349 PMC11511404

[12] Puerta Sarmiento GE, Modragón I, Echeverri A et al. Autoimmune/inflammatory syndrome induced by adjuvants (ASIA), medical treatment of severe systemic compromise: case report. Colomb Med (Cali) 2023;54:e5004625. 10.25100/cm.v54i1.4625 37424740 PMC10327631

